# Functional Assessment of Cancer Therapy-Brain questionnaire: translation and linguistic adaptation to Brazilian Portuguese

**DOI:** 10.1590/S1516-31802011000400006

**Published:** 2011-05-05

**Authors:** Mariana Rodrigues Gazzotti, Marcela Batan Alith, Suzana Maria Fleury Malheiros, Milena Carlos Vidotto, José Roberto Jardim, Oliver Augusto Nascimento

**Affiliations:** I PT, PhD. Coordinator, Neurosurgery Physiotherapy Research Group, Respiratory Division, Universidade Federal de São Paulo (Unifesp), São Paulo, Brazil.; II PT, MSc. Research Fellow, Neurosurgery Physiotherapy Research Group, Respiratory Division, Universidade Federal de São Paulo (Unifesp), São Paulo, Brazil.; III MD, PhD. Associate Professor, Neurology Division, Universidade Federal de São Paulo (Unifesp), São Paulo, Brazil.; IV PT, PhD. Associate Professor, Physiotherapy Department, Universidade Federal de São Paulo (Unifesp), São Paulo, Brazil.; V MD, PhD. Associate Professor, Respiratory Division, Universidade Federal de São Paulo (Unifesp), and Director of the Pulmonary Rehabilitation Center at Unifesp/Lar Escola São Francisco (Lesf), São Paulo, Brazil.; VI MD, PhD. Attending Physician, Respiratory Division, Universidade Federal de São Paulo (Unifesp), and Vice-Director of the Pulmonary Rehabilitation Center at Unifesp/Lar Escola São Francisco (Lesf), São Paulo, Brazil.

**Keywords:** Quality of life, Translations, Reproducibility of results, Brain neoplasms, Questionnaires, Qualidade de vida, Tradução (produto), Reprodutibilidade dos testes, Neoplasias encefálicas, Questionários

## Abstract

**CONTEXT AND OBJECTIVE:**

Quality of life assessment among patients with brain tumors is important, given that new treatments have increased patient survival. The aim of this study was to translate the Functional Assessment of Cancer Therapy-Brain (FACT-Br) questionnaire (version 4) into Portuguese, carry out cross-cultural adaptation and assess its reproducibility.

**DESIGN AND SETTING:**

Cohort at the Universidade Federal de São Paulo (Unifesp).

**METHODS:**

Forty patients with a brain tumor seen at the neuro-oncology outpatient clinic participated in the study. The process of translation and back-translation was carried out, along with adaptation to the Portuguese language and Brazilian culture. The intraclass correlation coefficient (ICC) was used to test the reproducibility of the FACT-Br (version 4).

**RESULTS:**

The reproducibility of the questionnaire was excellent (ICC = 0.95; 95% confidence interval, CI: 0.89-0.97). The ICC with a mean interval of 15 days between applications of the questionnaire was very good in all domains (ICC = 0.87 to 0.95). The mean time taken to answer the questionnaire was 6.27 ± 2.26 minutes, ranging from 3 to 11 minutes.

**CONCLUSION:**

The translated version of the FACT-Br questionnaire (version 4) adapted to the Portuguese language and Brazilian culture proved to be easily understood and achieved very good reproducibility among patients with diagnoses of brain tumors.

## RESUMO

**CONTEXTO E OBJETIVOS:**

A avaliação da qualidade de vida dos pacientes com tumor cerebral é importante, considerando que novos tratamentos têm aumentado sua sobrevida. O objetivo deste trabalho foi traduzir o questionário Functional Assessment of Cancer Therapy-Brain (FACT-Br) (versão 4) para a língua portuguesa, realizar a adaptação cultural e avaliar sua reprodutibilidade.

**DESIGN E LOCAL:**

Estudo de coorte realizado na Universidade Federal de São Paulo (Unifesp).

**MÉTODO:**

Quarenta pacientes com tumor cerebral provenientes do Ambulatório de Neuro-oncologia participaram do estudo. O processo de tradução inicial e retrógrada foi realizado, além da adaptação cultural para a língua e cultura brasileira. O coeficiente de correlação intraclasse (CCI) foi utilizado para testar a reprodutibilidade do FACT-Br (versão 4).

**RESULTADOS:**

A reprodutibilidade do questionário foi excelente (CCI = 0,95; intervalo de confiança, IC 95% 0,89-0,97). O CCI com intervalo médio de 15 dias entre aplicações do questionário, em todos os domínios, foi muito bom (CCI = 0,87 a 0,95). O tempo médio de resposta ao questionário foi de 6,27 minutos ± 2,26 minutos, variando de 3 minutos a 11 minutos.

**CONCLUSÃO:**

A tradução e a adaptação à língua e à cultura brasileira do questionário FACT-Br (versão 4) mostrou-se de fácil entendimento e com muito boa reprodutibilidade em pacientes com diagnóstico de tumor cerebral.

## INTRODUCTION

The incidence of central nervous system tumors in Brazil was found to range from 5.8 to 8.4 cases per 100,000 inhabitants among men and from 4.9 to 7.1 cases among women in 2007.^1^ These tumors varied in malignancy, but even the so-called benign tumors can have high morbidity and mortality rates, depending on their location. Moreover, some histological types of benign tumors may develop into malignant tumors.^2,3^

Patients with a brain tumor may experience intense changes in quality of life due to frequent headaches, anorexia, nausea, seizures and insomnia.^4–6^ These patients can also experience neurological deterioration, such as motor impairment, personality changes, cognitive impairment, aphasia or visual impairment, which have a considerable impact on their quality of life.^3,7,8^ Furthermore, the usual treatment normally involves surgery, radiotherapy and chemotherapy, as well as the frequent use of medications such as antiepileptic drug agents and corticosteroids, with an evident negative impact on quality of life.^9^

According to the World Health Organization, the concept of health is not merely the absence of disease, but rather, the individual perception of complete physical, mental and social wellbeing. Assessing health and the effects of treatment implies evaluating changes in the frequency and severity of the disease, as well as estimation of wellbeing. One of the ways to assess wellbeing is through quality-of-life questionnaires. Tools for measuring quality of life provide a useful way to transform subjective measurements into objective data that can be quantified and analyzed. Such tools are important for determining the impact of interventions on patients’ health and quality of life.^10^

Thus, the issue of quality of life may be looked at from different perspectives, going from a scientific perspective to an objective point of view but passing through the common-sense and subjective perspectives. When looked at from a broader perspective, it is clear that quality of life is composed of all the fundamental human spiritual and material necessities. In this sense, quality of life is not just an absence of disease: it always has to be regarded as an issue of public health care.^11^ The use of quality-of-life questionnaires is the way in which this issue has been commonly approached.

One of the most used quality-of-life questionnaires designed for patients with brain tumors is the Functional Assessment of Cancer Therapy-Brain (FACT-Br), which contains subscales that address physical, social/family, emotional and functional wellbeing, as well as any additional concerns. FACT-Br is a simple, short, self-administered questionnaire that was originally drafted and validated in the English language by Weitzner et al.^12^ To date, no questionnaire on brain tumors has been translated into Portuguese with cultural adaptation to Brazilian patients, thereby making it difficult to fully evaluate such patients.

## OBJECTIVE

The aim of the present study was to translate the FACT-Br questionnaire (version 4) into the Portuguese language as spoken in Brazil, carry out the necessary cross-cultural adaptation and assess its reproducibility.

## METHODS

### Type of study

This was a cohort study.

### Sample

Forty clinically stable patients with a diagnosis of a brain tumor from the Neuro-Oncology Clinic at the Universidade Federal de São Paulo (Unifesp) completed the present study.

All consecutive patients over 18 years of age with a histologically proven brain tumor who presented a minimum score of ≥ 24 for literate or ≥ 13 for illiterate individuals in the Mini-Mental State Examination (MMSE)^13^ and were not undergoing chemotherapy/radiotherapy treatment were included. Patients who were hospitalized during the evaluation process and those who did not show up for all evaluations were excluded. All participants signed an informed consent statement and the study received approval from the institution's Ethics Committee.

### Questionnaire structure

The FACT-Br contains 50 items addressing five aspects of quality of life: physical wellbeing, social/family wellbeing, emotional wellbeing, functional wellbeing and any additional concerns. The physical, social/family and functional wellbeing subscales each have seven items. The emotional wellbeing subscale has six items and the additional concerns subscale is made up of 23 items. There are five response options for each item, with scores ranging from 0 to 4 points.

### Translation into Portuguese and cross-cultural adaptation

The translation and cultural adaptation was carried out jointly with the functional assessment of chronic illness therapy (FACIT) measurement system in the United States, with all guidance determined by FACIT organization, which has the authorial rights to the questionnaire.

Firstly, two local translators were recruited. Both were native Portuguese speakers who were fluent in English. Each of them individually produced a version of the questionnaire in Portuguese. In the second step, a third native Portuguese-speaker fluent in English evaluated the two translated versions and consolidated them into a single version. This version of the questionnaire was then back-translated into English. This translation process was similar to that used for the translation of the Saint George Questionnaire for Respiratory Disease^14^ and the Airways Questionnaire (AQ 20).^15^

The third step consisted of evaluation by four bilingual staff members of the FACIT organization, who analyzed and asked questions regarding the first two translations and the consolidated translation. They asked for evaluations by three additional reviewers: one from the measurement system itself (a native English speaker fluent in Portuguese) and two native Portuguese speakers who were fluent in English and living in Brazil. These two translators received the questionnaire containing all the previous steps as well as an instruction guide that pointed out the differences in the previous translations.

The fourth step consisted of evaluation of the three versions from the third step, with discussion and correction by the FACIT organization, thereby generating a Portuguese version of the questionnaire for use among patients. The questionnaire was applied at interviews with 10 patients who were being followed up at the Unifesp Neuro-Oncology Clinic. The aim of the interviews was to assess the difficulties that patients might have in understanding the questionnaire and to determine the patients’ interpretations in all the subscales. The questionnaires and interviews were then sent to FACIT, together with a report from the principal researcher that explained the main difficulties faced by the patients, so that the final corrections could be made and the final version of the questionnaire could be written.

### Evaluation of the reliability of the FACT-Br questionnaire

To evaluate the reliability of the FACT-Br questionnaire (final version), it was given to 30 patients. Each patient filled out the questionnaire on three separate occasions. Only two researchers participated in distributing the questionnaires. The two researchers were taught to read out the questionnaire slowly and clearly, and it was established that the questions should not be interpreted for the patients. The questionnaire was first administered by two independent researchers at different times (Researchers 1 and 2). This procedure was used to determine the inter-observer reliability. After 15 days, it was again administered by Researcher 1 in order to determine the intra-observer reliability. The questionnaire was read out to all patients.

### Statistical analysis

Descriptive statistical analysis was used to demographically and clinically characterize the patients. Student's t-test was used to compare pairs of independent samples. The intraclass correlation coefficient (ICC) was used to measure reliability and 95% confidence intervals (95% CI) were determined. The significance level was set at P < 0.05. All statistical analyses were performed with the aid of the Statistical Package for the Social Sciences, version 17.0.

## RESULTS

Forty-seven patients with a diagnosed brain tumor were initially evaluated. Five were not included because they presented scores below the cutoff points for the MMSE; one was excluded due to a stroke during the study period; and one was excluded because of failure to return for the second visit. Thus, a sample of 40 patients completed the study, of whom 10 participated in the translation and cross-cultural adaptation phase and 30 participated in the reliability study. The patients remained clinically stable and their treatment remained unaltered over the 15-day interval between the times of administration of the questionnaire. All items of the questionnaires were filled out completely by all patients.

Among the 10 patients who participated in the translation and cultural adaptation phase, five (50%) were female. These patients’ mean age was 45.2 ± 11.4 years (Table 1). Among the 30 patients in the reliability study, 11 (36.7%) were female. Table 1 displays the patients’ characteristics. Patient cognition was assessed by means of the MMSE questionnaire; the minimum score was 13 for one illiterate patient and 24 for two patients with reading and writing skills. The mean time taken to apply the questionnaire was 6.27 ± 2.26 minutes, ranging from three to 11 minutes.

**Table 1.** Demographic data on the brain tumor patients

Variables	n = 10 translation and cultural adaptation	n = 30 reliability study
Age (years)	45.2 ± 11.4	40.20 ± 13.4
Gender (male)	5 (50%)	19 (63.3%)
Education		
Illiterate	1 (10%)	2 (6.7%)
< 10 years	4 (40%)	6 (20%)
> 10 years	5 (50%)	22 (73.3%)
Current treatment		
Corticosteroids	2 (20%)	4 (13.3%)
Anticonvulsant	5 (50%)	19 (63.3%)
Mini-mental state examination	27.1 ± 2.4	26.9 ± 3.3
Hemispheric side of the tumor		
Right	5 (50%)	13 (43.3%)
Left	4 (40%)	14 (46.7%)
Other	1 (10%)	3 (10%)
Prior treatment		
Chemotherapy	3	1
Radiation	1	8
Surgery	7	23
Chemotherapy and radiation	3	9

The intraclass correlation coefficient (ICC) from analysis on the intra-observer reliability (15-day interval between administrations) was 0.95 (95% confidence interval, CI: 0.89-0.97). The ICC from analysis on inter-observer reliability (two observers on the same day) was 0.95 (95% CI: 0.89-0.97).

The intraclass coefficients from the intra-observer reliability analysis on the different subscales (15-day interval) ranged from 0.87 to 0.95 (Table 2). Likewise, the intraclass coefficients from the inter-observer reliability analysis on the different subscales (two independent observers) also ranged from 0.87 to 0.95. These values were considered to be very good (Table 2). In the evaluation on subscale reproducibility, comparisons between the mean scores on the five subscales when the questionnaire was administered on separate occasions by the same researcher (Table 3) and on the same day by two independent researchers (Table 4) did not show any statistically significant differences. Moreover, the five subscales presented excellent internal consistency, with Cronbach's alpha ranging from 0.84 to 0.93.

**Table 2.** Intra and inter-observer reproducibility for each domain and scale of the Functional Assessment of Cancer Therapy-Brain (FACT-Br) questionnaire (version 4)

	Intra-observer	Inter-observer
	ICC* (95% CI)	ICC* (95% CI)
Physical wellbeing	0.92 (0.82-0.96)	0.91 (0.82-0.96)
Social/family wellbeing	0.95 (0.90-0.97)	0.95 (0.89-0.98)
Emotional wellbeing	0.95 (0.89-0.97)	0.87 (0.72-0.93)
Functional wellbeing	0.87 (0.72-0.94)	0.90 (0.79-0.95)
Additional concerns	0.93 (0.84-0.96)	0.89 (0.78-0.95)
Total score	0.95 (0.89-0.97)	0.95 (0.89-0.97)

ICC = intraclass correlation coefficient; CI = confidence interval;

* P < 0.05.

**Table 3.** Mean scores for each domain and scale of the Functional Assessment of Cancer Therapy-Brain (FACT-Br) questionnaire (version 4)

	V1 Mean ± SD	V2 Mean ± SD	Δ	P
Physical wellbeing	22.4 ± 4.2	22.2 ± 4.7	0.233	0.62
Social/family wellbeing	17.1 ± 6.4	17.6 ± 5.7	-0.513	0.28
Emotional wellbeing	18.9 ± 4	19.3 ± 3.9	-0.367	0.25
Functional wellbeing	19.3 ± 5.0	18.8 ± 5.9	0.467	0.50
Additional concerns	51.6 ± 10.7	52.0 ± 12.4	-0.500	0.66
Total score	129.3 ± 24.0	129.9 ± 27.8	-0.680	0.75

V1 = first visit; V2 = second visit; Δ = V1 – V2; SD = standard deviation.

**Table 4.** Inter-observer mean scores for each domain and scale of the Functional Assessment of Cancer Therapy-Brain (FACT-Br) questionnaire (version 4)

	Observer 1	Observer 2	Δ	P
Physical wellbeing	22.4 ± 4.2	22.3 ± 4.9	0.133	0.78
Social/family wellbeing	17.1± 6.4	17.2 ± 6.9	-0.127	0.81
Emotional wellbeing	18.9 ± 4	19.2 ± 4.2	-0.300	0.57
Functional wellbeing	19.3 ± 5.0	19.7 ± 5.2	-0.400	0.48
Additional concerns	51.6 ± 10.7	52.4 ± 10.9	-0.900	0.46
Total score	129.3 ± 24.0	130 ± 26.4	-1.593	0.43

Δ = Observer 1 – Observer 2.

## DISCUSSION

The lack of quality-of-life assessment tools that have been translated and adapted for the Portuguese language in Brazil, for patients with brain tumors, has restricted research in this field. It was decided to translate, culturally adapt and assess the reliability of the FACT-Br questionnaire because this tool specifically assesses the impact of brain tumors on quality of life. The methodology used made it possible to achieve an adequate translation of the original questionnaire, thereby enabling its use in assessments on Brazilian patients with a brain tumor.

The intra-observer and inter-observer reproducibility of the FACT-Br questionnaire were determined, and the ICC calculations demonstrated excellent agreement^16^ between administrations of the questionnaire by a single investigator on separate occasions as well as by two different investigators. For an assessment tool that analyzes patients’ conditions to be considered adequate for the scientific community, it must be reproducible.^17^ The minimum acceptable ICC value for demonstrating that an assessment measurement is reliable is greater than or equal to 0.70 when the questionnaire is new and 0.80 when the questionnaire is old.^18^ In the present study, the ICC values were greater than or equal to 0.87, thereby demonstrating excellent reliability. Moreover, there were no statistically significant differences between the mean values for each subscale at the two different evaluation times, thereby revealing very good reliability for the questionnaire as a whole as well as for each of its parts.

The five subscales exhibited excellent internal consistency, with Cronbach's alpha values that were higher than those of the original questionnaire.^12^ The highest value was in the additional concerns subscale, which was also the case for Weitzner et al.^12^ with the original questionnaire. This subscale may be considered to be the most important one, given that it specifically addresses the brain tumor. The other subscales address quality of life in general and make up part of the FACT-G questionnaire,^19^ which is used to assess quality of life with regard to different types of tumors.

There were changes in some of the responses after the 15-day interval, from the first administration of the questionnaire. This may have been because the questionnaire includes specific items relating to feelings that may change over a two-week period, such as “I feel sad”, or relating to physical issues such as “I have pain”. Another important point to take into account is the response options, which often have little difference between them, such as “quite a bit” and “very much”. In the first interview, the response may be “quite a bit”, and this may change 15 days later to “very much”. Clinically, this change may not be significant, while the two responses analyzed separately are computed as a change that has occurred.

The translation and cultural adaptation of the questionnaire complied with the guidance from the FACIT organization, which has particular features in comparison with other translation processes.^20,21^ After the back translation, the questionnaire was sent to FACIT in the United States for assessment by four bilingual individuals, and it was subsequently sent to three more reviewers in order to draft a report on the entire translation process. FACIT evaluated all the versions and generated a version to be administered in a pilot study with 10 patients. During this step, an interview was also conducted to assess the degree of understanding and/or difficulty that patients may have had when reading the items. The principal researcher then made a report addressing the main difficulties that the patients had and sent it to FACIT so that the final version could be developed. This broad-scope process with different translation and adaptation steps made it possible to achieve linguistic equivalence for words from the source language in the target language. Obviously, certain problems had to be addressed, as in the case of the word *seizure*. From a semantic standpoint, the word *seizure* (for which the literal translation is “ataque”) was a good example of how a difference between the source and target languages can distort the meaning from a cross-cultural standpoint. In the first version, the word *seizure* was translated as “ataque (convulsão)”; however, all the patients suggested that this should be changed to the term “crise convulsiva (convulsão)”.

For the reliability study, the two researchers involved in administering the questionnaire received specific training, which made the administration process homogeneous. Self-administered questionnaires should be simple and objective and should not raise doubts among patients. When a questionnaire fulfills these requirements, the need for training regarding its administration is minimal and thus it does not create confounding factors. In this study, both reliability results were considered to be excellent.^16^

The demographic characteristics regarding gender, age, schooling, tumor site and patient treatment in the present study were similar to those found in the original study for the FACT-Br questionnaire.^12^

Because 10 out of the 30 patients were illiterate or had a low level of schooling, we decided to read out the questionnaire to all the patients for the sake of uniformity. This questionnaire presents a capacity for interviewers to read it out to patients, and this is of fundamental importance for its administration in Brazil, given that 40% of the population is functionally illiterate.^22^

The translation and cultural adaptation of the FACT-Br questionnaire provides a tool that can be used in future studies on patients with brain tumors for specific assessment of quality of life, which is a parameter that has been underevaluated in neuro-oncology. Thus far, the majority of studies have used the Karnofsky scale to assess general wellbeing and the Barthel index to assess functional capacity,^23,24^ but these scales are not capable of assessing the quality of life of patients with a diagnosed brain tumor.^25^

## CONCLUSION

In conclusion, this version of the FACT-BR questionnaire (version 4), translated into Portuguese and adapted to Brazilian culture, proved to be patient-friendly and achieved very good reliability, thereby enabling its efficient use among Brazilian patients with a diagnosed brain tumor and enabling assessment for specific treatments.
